# Editorial: Understanding the female reproductive microbiome in livestock

**DOI:** 10.3389/fmicb.2025.1570990

**Published:** 2025-02-18

**Authors:** Nicholas Wege Dias

**Affiliations:** Department of Animal Sciences & Industry, Kansas State University, Manhattan, KS, United States

**Keywords:** reproductive microbiome, livestock, female reproduction, reproductive efficiency, livestock reproductive microbiome

Reproductive efficiency in livestock species is one of the main aspects of animal husbandry to allow sustainability of food production. To support the efficiency of animal production systems, a dam must endure several reproductive challenges, such as early puberty and pregnancy, giving birth without difficulties, weaning healthy offspring, and rebreeding in a timely manner. Over the past decades, significant advancements have been made in livestock reproduction that have provided a comprehensive understanding of the physiological processes involved in the estrous cycle, fertilization, and pregnancy. These advancements have enabled the development of various reproductive technologies that can be adopted in livestock systems and promote production efficiency. While knowledge of female reproductive physiology is well advanced, the science of the reproductive microbiome in livestock species is still in its early stages and advancements in this area will allow for continued improvement of reproductive efficiency in agriculture.

The current Research Topic (RT) focuses on the reproductive microbiome and the features of the gastrointestinal microbiome associated with reproduction in livestock species such as the cow, sow, and ewe. This RT encompasses the establishment of the reproductive microbiome following artificial insemination, the reproductive microbiome during gestation, and features of the microbiome related to birth complications and the postpartum period. Included here is a review by Dias et al. that explores the dynamic interactions between the host and the microbiome, providing insights into the role of microbiota in promoting homeostasis and protecting the host from disease, while highlighting findings from the rich literature on the human vaginal microbiome as a template for reproductive microbiome studies and reviewing the literature on the bovine vaginal microbiome, exploring its relationships with fertility and reproductive disease.

It was previously believed that the uterus was a sterile environment and that introduction of microorganisms in this organ would result in pregnancy complications and reproductive disease. This concept of a sterile uterine environment has been challenged by recent studies. Featured in this RT, Moraes et al. (a) explored whether the bovine uterine microbiome is established in virgin heifers following artificial insemination, a commonly used reproductive technology, or naturally through the acquisition of microbiota from the vagina and the cervix. In cattle operations, estrous synchronization protocols (ES) are commonly adopted prior to artificial insemination to synchronize ovulations, thus enabling timed artificial insemination (TAI) to be performed on a large group of cows within the same day. Smith et al. explored both the change in vaginal microbiome composition, and the blood cytokine profile of *Bos indicus* beef cows during an ES and its relationship with conception rates to TAI. While achieving great conception rates is desirable in livestock systems, pregnancies must be sustained to term, and complications during gestation can lead to pregnancy complications and abortion. In this RT, Cassas et al. longitudinally characterized the ewe vaginal microbiome throughout gestation by 16S rRNA sequencing, and Moraes et al. (b) characterized the microbiome of several sites from the pregnant uterus and fetal membranes in Holstein dairy cows and heifers following slaughter by both sequencing and culture methods.

Finally, complications during parturition, such as dystocia, pelvic organ prolapse, and postpartum reproductive diseases can compromise the reproductive efficiency of livestock systems, and not only affect offspring and the dam's ability to survive, but also the ability of the dam to rebreed in a timely manner. Kiefer et al. categorized pregnant sows according to pelvic organ prolapse risk and characterized the fecal microbiome composition during late gestation and its correlations with the vaginal microbiome and pelvic organ prolapse. Furthermore, Liu et al. explored the structure and function of the microbiome at several gastrointestinal sites and its relationship with energy metabolism and blood metabolite composition in dairy cows during the postpartum period, providing insights into temporal changes in metabolism during the postpartum period, which could inform novel dietary and management strategies during the postpartum period. Finally, Guo et al. utilized the lactic acid producing bacteria, *Lactobacillus johnsonii* isolated from uterine secretions of healthy dairy cows to engineer a recombinant *Lactobacillus johnsonii*, expressing bovine granulocyte-macrophage colony-stimulating factor, and utilized a specific pathogen-free mice model to explore the effects of inoculating these strains during late-gestation in protecting against uterine inflammation following a postpartum *Escherichia coli* challenge.

The studies featured in this RT encompass significant scientific contributions from several authors to the knowledge of the reproductive microbiome in livestock species, addressing a variety of reproductive processes that are relevant to livestock production, and highlighting the complex and important role the microbiome plays in reproductive health and efficiency.

